# Establishing a national indicator-based surveillance system for hospital bed utilization by COVID-19 patients in the Philippines

**DOI:** 10.5365/wpsar.2023.14.5.1038

**Published:** 2023-09-30

**Authors:** Bienvenido S Cabaro, Gabrielle Ann T Dela Paz, Jeffrey B Dotingco, Bernadette Joy Q Almirol, Gabriel R Borlongan, Reena Ophelia D Cebreros, Patrick B Diangco, Kenneth Pierre B Quijalvo, Joanne Pauline U Tan, Ramon Rafael D Tonato

**Affiliations:** a Health Facility Development Bureau, Department of Health, Manila, Philippines.; b World Health Organization Regional Office for the Western Pacific, Manila, Philippines.; c Field Implementation and Coordination Team, Department of Health, Manila, Philippines.; d Knowledge Management and Information Technology Service, Department of Health, Manila.

## Abstract

In March 2020, the Philippine Department of Health (DOH) designed and rapidly implemented a national surveillance system for the utilization of hospital beds by patients with coronavirus disease (COVID-19) to produce complete and timely data for use by various levels of governance in response to the COVID-19 pandemic. The DOH launched the DOH DataCollect (DDC) Bed Tracker system, a web-based application that collects information from all 1906 public and private hospitals and infirmaries across the country using a modular data collection tool. Data on the maximum number of occupied COVID-19-designated beds (*n* = 28 261), hospital bed utilization rate (71.7%), and mechanical ventilator number (*n* = 1846) and utilization rate (58.5%) were recorded in September 2021 during the Delta surge of cases in the Philippines. Data on human resources, personal protective equipment and supplies, and other operational indicators were added to the system during various modifications. Information from the DDC was used to inform the COVID-19 response and operations at national and local levels and facilitated research at academic and nongovernmental agencies. The development of the DDC system demonstrates that an effective surveillance system for use by all health-care facilities is achievable through strong national leadership, the use of available technology and adaptive information systems, and the establishment of networks across different health facilities and stakeholders.

The first case of coronavirus disease (COVID-19) in the Philippines was confirmed on 30 January 2020. By 1 March 2020, there were 633 suspected COVID-19 cases admitted to hospitals across the country. (*1*) Given the increasing number of cases and the infectious nature of the disease, (*2*) data on hospital admissions were vital for health system policies and decision-making for the COVID-19 response, (*3*) including health facility operations, patient referrals, and public health and social measures.

Prior to the COVID-19 pandemic, health information systems (HISs) in the Philippines were fragmented; there was a lack of IT infrastructure in health facilities and a devolved health system, with some hospitals managed by the national government and others by local governments. With the sudden increase in COVID-19 cases in February 2020, health facilities needed guidance through government policies to address inconsistencies, untimeliness and poor quality of data submissions. Data collection methods included consolidated spreadsheets from health facilities and daily enquiries about their hospital bed utilization rates. There were no standardized processes, no prior data cleaning and no validation of submissions. In March 2020, the demand for data on hospital beds and medical equipment increased, but existing systems were unable to provide hospital admissions data to decision-makers.

The urgent need for hospital admissions data at this time exposed the vulnerability of the HIS and the lack of routine surveillance systems, especially on health emergencies and health facility capacity. Also, some existing registries and information systems for active surveillance of specific diseases and health events were poorly integrated from the local to national level. (*4*) Previous attempts to create a health emergency preparedness and response information system to improve the government’s action and response during emergencies (*5*) had not materialized. With these fragmented systems, policy- and decision-makers did not have access to the information they needed and, therefore, had to rely on ad hoc data collection.

There was an urgent need to establish a national indicator-based surveillance system to gather timely and accurate information on the capacity of health-care facilities for COVID-19 patients and to project demands for resources. These data were vital to informing key responses and operations on COVID-19. (*6*) On 3 March 2020, the Philippine Department of Health (DOH) DataCollect (DDC) Bed Tracker system was launched to regularly receive data from all public and private hospitals and infirmaries in the country on their health resource availability and needs. (*3*) This paper describes the establishment of the DDC system and how it was used during the Philippines’ COVID-19 response.

## Methods

There were four stages in the establishment and improvement of the DDC system for COVID-19 hospitalizations in the Philippines, which started in February 2020 and are still ongoing. A team of policy-makers, physicians, data analysts and IT developers in the DOH was assigned to lead and perform the continuous development, analysis, report generation and dissemination of the DDC system. A network of regional officers and hospital data entry officers was also formed to ensure proper implementation and regular monitoring of the system on the ground.

### Stage 1: planning

The main objective of the DDC system was to monitor the occupancy rate of COVID-19-designated beds and equipment in all public and private hospitals and infirmaries in the Philippines. The DOH designed an application programming interface (API) that gathered information on COVID-19 and non-COVID-19 bed utilization from all hospitals and infirmaries across the country. This API referenced the National Health Facility Registry (NHFR) to create user accounts for the facilities to access during the data collection process. It had several initial indicators and monitoring questions **(Box 1)**.

### Stage 2: implementation

The DDC system (https://hfpddc.doh.gov.ph), originally a mobile application, was launched on 3 March 2020. The DOH issued Department Memorandum 2020–0136, dated 25 March 2020, and Department Circular 2020–0158, dated 27 March 2020, mandating all hospitals and infirmaries to submit reports daily, weekly and as needed. (*3*) The facilities’ data entry officers submitted the required data (**Box 1**) through the DDC system’s API, which were then stored in the DOH data warehouse.

**Fig. 1:**
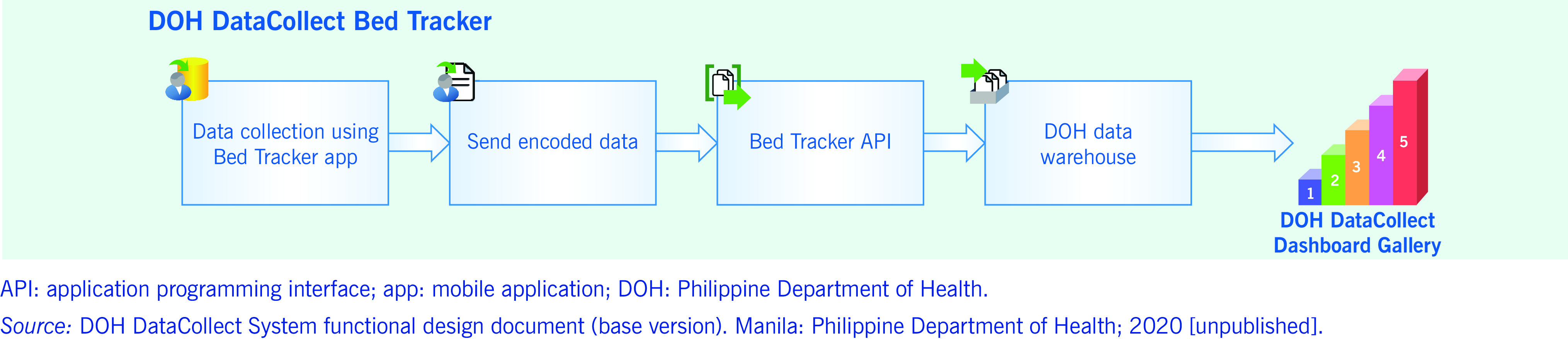
Data flow of the DOH DataCollect Bed Tracker System

Orientation sessions for facility encoders were conducted before the start of the DDC system and before each update was implemented. The DOH and its regional officers constantly provided technical support to facility encoders regarding DDC processes, interfaces and tools. The regional officers also conducted periodic reviews of DDC questions and indicators, monthly monitoring of submissions to ascertain compliance, and quick assistance in validating flagged entries in the system. Facilities were required to submit incident reports to document any corrections in data submissions. Correspondingly, identified errors were rectified by the DOH through direct and documented editing of the data warehouse. The validated data were then extracted to update dashboards and create daily reports (**Fig. 1**).

[**insert** Figure 1]

### Stage 3: data processing, analysis and dissemination

The data gathered from the DDC system underwent automated data processing, which includes deduplication and merging of variables stored in the NHFR such as geographic information, facility service capabilities, ownership, and the number of beds. The data were then analysed to create detailed internal reports such as weekly health facility capacity reports, which were disseminated to the DOH executive, technical and regional offices, and other national government agencies for decision- and policy-making. The data were also available through the DOH’s open-access database called DataDrop, with internal and public-facing dashboards, and used for public information materials for the official country COVID-19 bulletin and reports. (*7*) The data were also used for academic research and analyses by nongovernmental agencies.

### Stage 4: iterations

The dynamic demand for information changed over the course of the pandemic. Therefore, the DDC questions and indicators were continuously updated by the DOH according to the needs of decision- and policy-makers, prevailing guidance from stakeholders, reviews of related information systems used internationally, and feedback from key informants. Lessons identified from previous DDC system versions were also used to refine the implementation and resource materials, including the tools, report templates and dashboard designs of succeeding DDC versions.

## Results

The first version of the DDC system was released with four variables being collected weekly. The 10th version, launched on 26 August 2022, has variables collected on a daily and weekly basis. The data completeness from 1906 public and private hospitals and infirmaries reached 80–95% by the third version of the DDC system, released in April 2020, and has improved to 98% as of December 2022 (**Table 1**). This shows how the system gained acceptability from health facilities as the immediate feedback and response from the government was highly valuable.

**Fig. 2:**
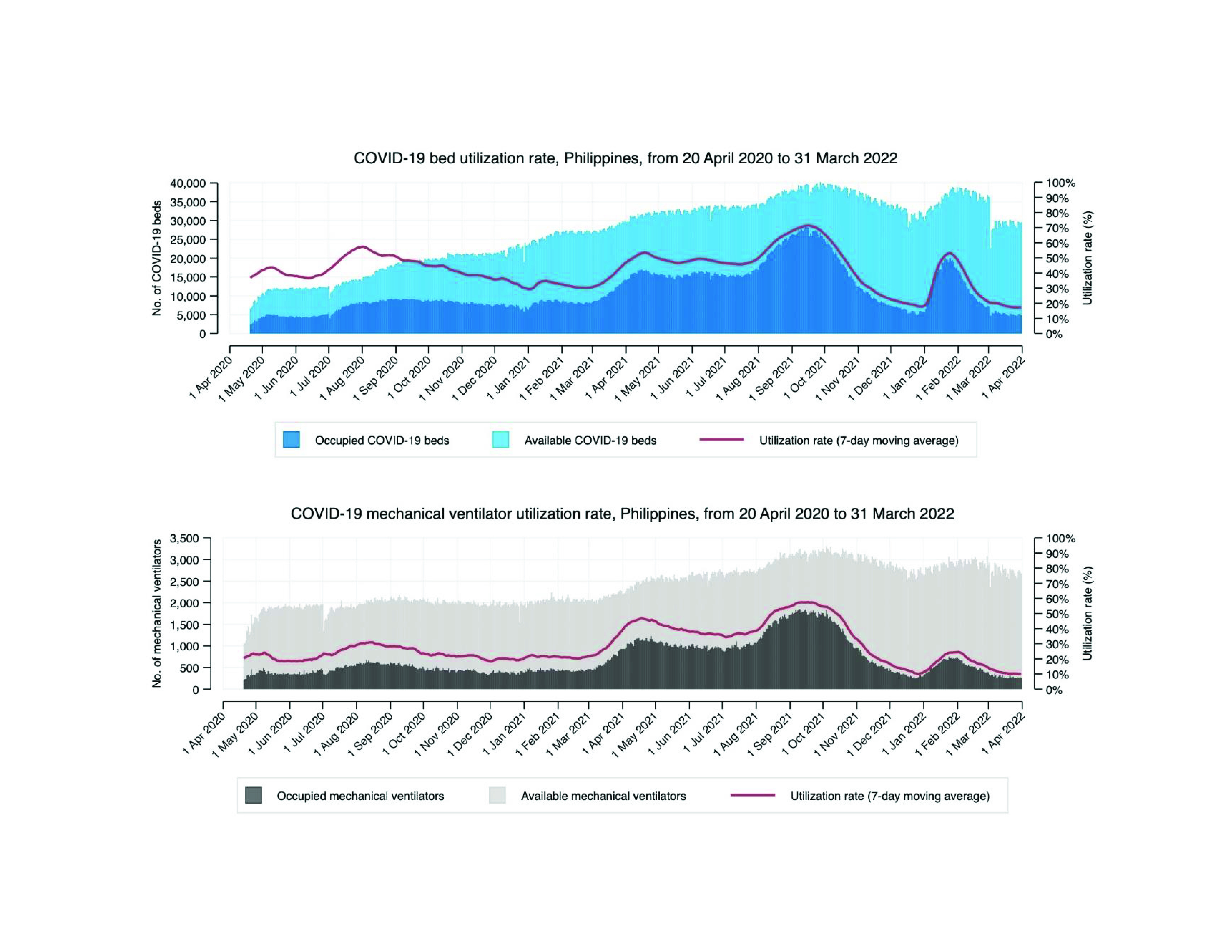
Utilization rates of COVID-19-designated hospital beds (a) mechanical ventilators (b), the Philippines, 20 April 2020 to 31 March 2022

The DDC system provided the number of occupied COVID-19-designated beds and the corresponding occupancy rates (**Fig. 2**). The maximum number of occupied COVID-19-designated beds of 28 261 and the maximum utilization rate of 71.7% were recorded in September 2021 during the country’s COVID-19 Delta surge (**Fig. 2**). The number of mechanical ventilators in use by COVID-19 patients also peaked in September at 1846 units, with a utilization rate of 58.5% (**Fig. 2**).

[**insert** Figure 2]

The DDC data were used by the government and other stakeholders in the country for the following purposes:

Implementing the COVID-19 Alert Level System, which decides the quarantine status of each province in the country based on the health-care utilization rate.
Modelling and forecasting by the subtechnical working group analytics for COVID-19. The spread of COVID-19 infections was slowed and prevented by risk-based public health interventions (for example, mobility restrictions) implemented by the Philippine COVID-19 Inter-Agency Task Force for the Management of Emerging Infectious Diseases based on the health-care utilization rates. (*8*)
Determining geographic areas and hospitals with the highest percentage of unavailable human resources, hospitals lacking in supplies (for example, personal protective equipment, oxygen), and other commodities.
Avoiding the health system being overwhelmed (crossing the “red line”) by monitoring indicators on hospital resources, which informed stakeholders on the allocation of COVID-19 beds and equipment, health-care workers and supplies. (*9*)
Monitoring indicators on health-care workers’ infections that affected health facility operations during COVID-19 surges through implementation of infection prevention and control (IPC) protocols while oxygen shortage problems were addressed by increasing supply in areas with high medical oxygen consumption rates and critical care utilization rates during the surge of Delta and Omicron variants of COVID-19.
Informing daily operations and patient referrals in facilities as well as areas needing step-down care, and quarantine and isolation facilities. Prompt medical treatments were given to Filipinos needing hospital care using data on patient navigation and referral systems among health facilities through the establishment of the National Patient Navigation and Referral Center. Well planned procurement and efficient distribution of vaccines in the country used evidence-based COVID-19 vaccine effectiveness reports, which incorporated hospitalization data of vaccinated individuals in the DDC system.
The open sharing of data with the public, specifically researchers and think tanks from the academic community and the private sector, for their own review and analysis.

## Discussion

The DDC system and its corresponding contribution to health facility monitoring and surveillance systems overcame many HIS issues in the Philippines by standardizing, centralizing and digitizing data submission from health facilities to the DOH. The timely establishment of the DDC system provided these data from health facilities during the COVID-19 pandemic. Data timeliness, precision and ease of use were prioritized in the design of the system.

The success of the DDC system was a result of strong leadership, a dedicated and competent management team, a strong network of government units, an adaptive information system with proper design, and the innovative use of available technology. (*10*) The system had high-level political support, which helped produce the needed resources to develop the system and orchestrate its nationwide implementation. Laws and policies were introduced that mandated reporting by hospitals and infirmaries through the system. (*3*, *10*) The DDC system used existing networks for collaboration and coordination and had different units working together, with regional officers working between the national government and local governments.

The DDC system was planned and then modified based on current needs and situations following the “enter, store, process, communicate, and present” concept. (*11*) Data entered by all hospitals were automatically stored, processed and analysed for communication materials. These were presented to decision-makers to facilitate timely response including public health and social measures, strategic resource allocation, and local and facility-based operations. Furthermore, publishing the data from the DDC system demonstrated the DOH’s transparency, enabled data quality assurance as external stakeholders could provide feedback on the data, and facilitated research by academic and nongovernmental agencies.

The DDC system had two main limitations. First, the system did not collect real-time data for patient referrals, unlike nationally integrated electronic medical record (EMR) systems. Instead, it collected daily aggregated tallies per facility, which required data entry into the DDC system, even in hospitals with mature EMR systems. This challenge could be addressed by further improving hospital HISs by investing in IT infrastructure in the Philippines. Second, the responsiveness of the system to collect new indicators depends on decision-makers’ ability to anticipate their information needs.

The DDC system became the first online monitoring and surveillance system for daily health facility operations of all hospitals and infirmaries in the Philippines despite the challenges of a devolved health system. The system was easily accessible and did not require resource-intensive IT infrastructure. It had high response rates and timely reporting from health facilities. Due to the success of the DDC system, similar data collection applications were developed for the 11 000 COVID-19 isolation and quarantine facilities nationwide, as well as vaccination data (i.e. the COVID-19 Vaccination Quick Count). While originally designed for COVID-19 pandemic surveillance, the DDC system can be used to build an effective and long-term HIS for universal health-care monitoring. This includes plans to convert the DDC system into a modular profiling system for all health facilities in the country covering primary, secondary, tertiary and specialized levels of health care.

## Conclusion

The need for up-to-date information on bed utilization from health facilities during the COVID-19 pandemic led to the development of the Philippines’ DOH DataCollect Bed Tracker system. This indicator-based surveillance system provided data for evidence-based policies and tailored COVID-19 responses. Even with existing HIS challenges and the resource limitations of a lower-middle-income country, this timely, effective and responsive surveillance system was established through strong national leadership, appropriate expertise and management, teamwork, use of an adaptive information system with relevant surveillance design, and proper use of available technology. The success of the DDC system contributes to an integrated and responsive surveillance system for universal health care in the Philippines.
